# Rapid agitation control with ketamine in the emergency department (RACKED): a randomized controlled trial protocol

**DOI:** 10.1186/s13063-018-2992-x

**Published:** 2018-11-26

**Authors:** David Barbic, Gary Andolfatto, Brian Grunau, Frank X. Scheuermeyer, William MacEwan, William G. Honer, Hubert Wong, Skye P. Barbic

**Affiliations:** 1 Department of Emergency Medicine, St Paul’s Hospital, 1081 Burrard St, Vancouver, BC V6Z 1Y6 Canada; 2 Department of Emergency Medicine, Lion’s Gate Hospital, 231 15th St E, North Vancouver, BC Canada; 3 Department of Psychiatry, University of British Columbia, Vancouver, BC Canada; 4 School of Public Health and Epidemiology, University of British Columbia, Vancouver, BC Canada; 5 Department of Occupational Science and Occupational Therapy, Vancouver, BC Canada; 6 Centre for Health Evaluation Outcome Sciences, Vancouver, BC Canada

**Keywords:** Ketamine, Agitation, Midazolam, Haloperidol, Randomized controlled trial, Emergency medicine

## Abstract

**Background:**

The rapid control of patients presenting to the emergency department (ED) with psychomotor agitation and violent behavior is paramount for the safety of patients and ED staff. The use of intramuscular (IM) ketamine in the pre-hospital and ED settings has demonstrated promising preliminary results to provide rapid and safe behavioral control. A prospective, randomized controlled trial is required to measure the potential superiority of IM ketamine compared to current standard care (IM benzodiazepines plus antipsychotics).

**Methods:**

This will be a parallel, prospective, randomized, controlled trial of 5 mg/kg IM ketamine compared to a combination of 5 mg IM midazolam and 5 mg IM haloperidol. The study will enroll approximately 184 patients, randomized equally to two study arms. There will be one study visit during which study medication will be administered and assessments will be completed. A follow-up safety visit will occur on day 3. The primary objective of this study is to compare IM ketamine to a combination of IM midazolam and haloperidol with regards to the time required for adequate behavioral control, in minutes, in patients presenting to the ED with psychomotor agitation and violent behavior, as measured by the Richmond Agitation-Sedation Scale (RASS).

**Discussion:**

We present a novel study to determine whether ketamine is a rapid and safe option, compared to a combination of midazolam and haloperidol for the sedation of patients presenting to the ED with psychomotor agitation and violent behavior. To our knowledge, this study is the first randomized controlled trial to compare ketamine to current standard care for this indication. We have attempted to address numerous logistical issues with the design of this study including a waiver of consent, ensuring adequate blinding of outcome assessors, patient enrolment, and data monitoring.

**Trial registration:**

Clinicaltrials.gov, NCT03375671. Registered on 18 December 2017.

**Electronic supplementary material:**

The online version of this article (10.1186/s13063-018-2992-x) contains supplementary material, which is available to authorized users.

## Background

The rapid control of patients presenting to the emergency department (ED) with psychomotor agitation and potentially violent behavior is paramount for the safety of patients and ED staff. Mental health crises, substance misuse, hypoxia, dementia, and metabolic or central nervous system disease processes are common reasons for agitation and potentially aggressive behavior in patients presenting to the ED [1]. Patients with this presentation provide a unique challenge to ED staff whose primary concerns are both patient safety and determining the cause of the agitation and violent behavior. Behavioral emergencies in the ED are multifactorial, complex, and require prompt response from ED staff to prevent injury of the patient, staff, and other visitors to the department. Although referred to as a “last resort,” an approach to control such situations in the ED is the rapid administration of intramuscular (IM) medications. This allows ED staff to assess, stabilize, diagnose, and monitor patients in a way that is safe for both patient and ED staff [1–4]. Three main classes of medications exist to restrain a patient in the ED: (1) benzodiazepines; (2) typical or classical antipsychotics; and (3) atypical antipsychotics. Ensuring patients receive the right intervention at the right time is critical to optimize the health and recovery outcomes of this vulnerable patient group. Emergency clinicians lack a clear, consensus standard of care as demonstrated by a recent systematic review on this topic [5].

Sedation of agitated and potentially violent patients in the ED with benzodiazepines is associated with an increased risk of respiratory depression, oxygen desaturation, and the need for airway interventions [5]. The use of antipsychotic medications for the sedation of agitated patients is associated with dystonia, akathisia, parkinsonism, and neuroleptic malignant syndrome [6].

Ketamine is a non-competitive N-methyl-D-aspartic acid receptor (NMDA) antagonist and a highly effective dissociative anesthetic [7]. Ketamine provides rapid dissociative anesthesia and analgesia and has been used extensively for a number of indications including low-dose analgesia [8, 9], procedural sedation [10–12], and general anesthesia in resource-limited settings [13]. Ketamine has many potential benefits for IM sedation of the agitated and violent patient, including [14] rapid dissociation to facilitate behavioral control and favorable cardiovascular stability compared to other agents used for sedation [15].

Ketamine preserves patients’ inherent respiratory drive, minimizing the risk of respiratory depression, oxygen desaturation, and other related adverse events (AEs) [11, 12, 16]. Ketamine provides analgesia for procedures required in the assessment and management of these patients, such as intravenous insertion and phlebotomy. A low rate of AEs with the use of ketamine for procedural sedation [7, 17] suggests that ketamine may be a safe medication for IM behavioral control of the agitated and violent patient in the emergency department setting.

The use of IM ketamine in the pre-hospital setting has been described in several retrospective chart reviews and prospective series [3, 18–26] and has been demonstrated to provide rapid and safe behavioral control [18, 23, 24]. These studies have also demonstrated favorable AE profiles. Retrospective chart reviews and prospective case series from the ED indicate effective and rapid sedation with ketamine [25, 26]. The main methodological limitations of these studies are their retrospective nature and the lack of a controlled comparison group. Furthermore, a recent position statement by the American College of Emergency Physicians highlighted the need for “high-quality research…to establish the safety and efficacy of ketamine compared with other agents for the control of the acutely agitated patient in the ED” [27]. As a result, a prospective, randomized controlled trial is required to measure the potential superiority of IM ketamine compared to current standard care (IM benzodiazepines plus antipsychotics) for the rapid and safe control of patients presenting to the ED with psychomotor agitation and violent behavior.

## Methods

### Overall study design

This will be a parallel, prospective, randomized, controlled trial of 5 mg/kg IM ketamine compared to a combination of 5 mg IM midazolam and 5 mg IM haloperidol.

The study will enroll approximately 184 patients, randomized equally to two study arms. There will be one study visit during which study medication will be administered and assessments will be completed. A follow-up safety visit will occur on day 3. The primary objective of this study is to compare IM ketamine to a combination of IM midazolam and haloperidol with regards to the time required for adequate behavioral control, in minutes, in patients presenting to the ED with psychomotor agitation and violence, as measured by the Richmond Agitation-Sedation Scale (RASS; Additional file 1). The Richmond Agitation Sedation Scale is a validated, 10-level sedation scale (+ 4 “combative” to − 5 “unarousable”) used to quantify patient agitation [28].

### Primary objective

The primary objective of this study is to compare IM ketamine to a combination of IM midazolam and haloperidol with regards to the time required for adequate sedation, in minutes, in ED patients with psychomotor agitation and violent behavior.

### Secondary objectives

The secondary objectives of this study include:

1. Investigating the safety and tolerability of ketamine;
2. Evaluating the percentage of participants in each arm requiring rescue medications at 5–30 min (at 5 min intervals) after study medication(s) administration. Rescue medications are defined as benzodiazepines, antipsychotics, or other sedative medications prescribed by the treating emergency physician for the purpose of achieving behavioral control;
3. Evaluating the percentage of participants experiencing sedation outcomes in each arm as defined by the TROOPS criteria (Additional file 1);
4. Evaluate the percentage of participants in each arm with neuroleptic malignant syndrome within 24 h of enrolment, adjudicated to be due to the administration of study medications;
5. Describe the pre-hospital use of force by police in this patient population;
6. Evaluate the participants’ clinical study experience;
7. Evaluate the study ED nurses’ clinical study experience;
8. Evaluate the effectiveness of blinding in the study.

## Selection and enrolment of participants

### Number of participants

The study will enroll approximately 184 participants.

### Inclusion criteria

1. Age 19–60 years inclusive;
2. Patients presenting to the ED with psychomotor agitation or violent behavior (RASS score > + 3).

### Exclusion criteria

1. Age < 19 years;
2. Age > 60 years;
3. Previous participation in this study;
4. Women suspected or known to be pregnant or breastfeeding;
5. Previous known hypersensitivity, intolerance or allergy to ketamine, midazolam, or haloperidol or their components;
6. Individuals who are in comatose states or have central nervous system (CNS) depression due to alcohol or are taking other depressant drugs;
7. Individuals with severe depressive states, spastic diseases, and with Parkinson’s disease, except in the case of dyskinesias due to levodopa treatment;
8. Senile patients with pre-existing Parkinson-like symptoms;
9. Individuals with a history of cerebrovascular accident;
10. Individuals in whom a significant elevation of blood pressure would constitute a serious hazard, such as patients with significant hypertension;
11. Individuals with severe cardiac decompensation;
12. Individuals who intend to have surgery of the pharynx, larynx, or bronchial tree unless adequate muscle relaxants are used;
13. Individuals with acute pulmonary insufficiency;
14. Individuals with severe chronic obstructive pulmonary disease;
15. Individuals with acute narrow angle glaucoma.

### Enrolment procedures

Designated study staff in the ED will identify potential participants – adult patients who present to the ED with psychomotor agitation or violent behavior – by communicating directly with the study investigator or sub-investigator. Patients will be enrolled when study staff are available in the ED. The study will be conducted at St Paul’s Hospital ED.

A waiver for informed consent has been granted by the UBC Providence Health Care Research Ethics Board (PHC REB) for this study. In cases where a potential participant presents to the ED accompanied by a substitute decision-maker, informed consent will be obtained from the substitute decision-maker.

A substitute decision-maker must be either: (1) the participant’s spouse, child, parent sibling, grandparent, grandchild, or any anyone else related by birth or adoption to the individual; (2) a close friend of the participant; or (3) a person immediately related to the individual by marriage. Furthermore, the substitute decision-maker must be aged at least 19 years, have been in contact with the participant during the preceding 12 months, have no dispute with the individual, and be capable of giving, refusing, or revoking substitute consent on the participant’s behalf.

The substitute decision-maker must decide to give or refuse consent in the participant’s best interests. When deciding whether it is in the participant’s best interests to give or refuse substitute consent, the substitute decision-maker must consider the individual’s current wishes and known beliefs and values, whether the individual’s condition or wellbeing is likely to be improved by the proposed healthcare, whether the participant’s condition or wellbeing is likely to improve without the proposed healthcare, whether the benefit the individual is expected to obtain from the proposed healthcare is greater than the risk of harm, and whether a less restrictive or less intrusive form of healthcare would be as beneficial as the proposed healthcare.

## Withdrawal of participants

A participant may be withdrawn from the study due to the reasons listed below including, but not limited to:

- □ The participant’s or substitute decision-maker’s request;
- □ Severe adverse effects, serious adverse events (SAEs), and/or other safety reasons;
- □ It is in the participant’s best interest according to the Investigator’s clinical judgment;
- □ The Sponsor–Investigator terminates the study.

All premature discontinuations and their causes must be documented by the Investigator on the appropriate CRF pages, e.g., final status, AEs.

Participants are free to withdraw from the study at any time and for whatever reason, specified or unspecified, and without prejudice to his or her medical care by a physician.

If a participant withdraws or is removed from the study for any reason before the completion of the study, the reason for, and date of, the discontinuation, and the date of the last dose of the study medication must be recorded in the appropriate section of the CRF.

## Randomization and blinding procedures

### Randomization

The study biostatistician will generate the treatment allocations before the start of the study using a randomized block design with varying block sizes. Each block will contain equal numbers of participants for each arm. The randomization schedule will be stored with the study medications in the Omnicell medication storage system in the ED. The group allocation will be concealed from blinded study staff by a sealed, opaque envelope with a unique study identifier code on the exterior. This will be withdrawn from the Omnicell by a designated unblinded ED study nurse administering medications. The unblinded ED study nurse will prepare and administer study medication after opening the next sequential envelope in the randomization system stored with the study medication. Treatment allocation will be concealed from all other study investigators, study staff, and participants, following the administration of study medications.

### Blinding

Blinding occurs immediately following enrolment into the study and group allocation. All participants, investigators, and study staff conducting study procedures will be blinded to study group allocation. The ED nurse administering study medication will be the only unblinded individual involved with the study. This ED nurse is not involved with the assessment of any study outcomes or other procedures related to the study.

## Study treatments

The study medications and doses were chosen after a lengthy review of the existing literature [29] and a survey of regional ED physicians’ preferences for sedation of this patient population. Midazolam and haloperidol are medications widely used in emergency medicine for rapid behavioral control of the agitated and violent patient [5, 6]. These medications could reasonably be considered “standard therapy” for this indication by emergency physicians.

### Investigational product description

**Generic name:** ketamine hydrochloride injection.

**Brand name:** Ketalar®, DIN 00224405 (10-mL vial, 500 mg/10 mL).

Ketalar® is a rapid-acting, non-barbiturate general anesthetic. It produces an anesthetic state characterized by profound analgesia, normal pharyngeal-laryngeal reflexes, and normal or slightly enhanced skeletal muscle tone. Mild cardiac stimulation and occasionally respiratory depression occur. The anesthetic state produced by Ketalar® has been termed “dissociative anesthesia” in that it appears to selectively interrupt association pathways of the brain before producing somesthetic sensory blockage. Ketalar® decreases the activity of the neocortex and subcortical structures (thalamus) and increases the activity in the limbic system and reticular substance.

### Dosing and administration

Participants in the treatment arm will receive a single intramuscular injection of 5 mg/kg Ketalar® administered by an unblinded study nurse.

Potential side effects:

- □ Temporary augmentation of heart rate and blood pressure
- □ Hypotension, arrhythmias, bradycardia
- □ Respiratory depression (with IV administration only)
- □ Laryngospasm
- □ Increased salivation
- □ Enhanced skeletal muscle tone
- □ Blurred vision, nystagmus, diplopia (transient)
- □ Anorexia, nausea, vomiting
- □ Transient erythema, morbiliform rash, anaphylaxis
- □ Increased intraocular pressure (transient)
- □ Emergence reaction (hallucinations or frightening depersonalization)

### Comparative treatments

#### Midazolam

**Generic name:** Midazolam injection, USP.

**Brand name:** n/a; DIN 02242905 (5 mg/mL).

Midazolam possesses all the pharmacological effects of the benzodiazepines, namely it is a sedative, hypnotic, anticonvulsant, anxiolytic, muscle relaxant and amnestic agent. In addition, midazolam enhances GABAergic inhibition, decreases the firing rate of single neurons, decreases the cerebral metabolic rate for oxygen, decreases cerebral blood flow, enhances the survival time of mice in a hypoxic milieu, and induces amnesia in the passive avoidance paradigm.

Midazolam binds in nanomolar concentrations to the high-affinity, stereospecific, benzodiazepine receptor sites in the mammalian brain. These receptor sites are functionally coupled to GABA recognition sites and to sites related to chloride channels. Midazolam decreases the cyclic GMP level in the cerebellum.

### Dosing and administration

The control group will receive a single intramuscular injection of the combination 5 mg midazolam and 5 mg haloperidol administered by an unblinded study nurse.

Potential side effects:

- □ Increased or decreased mean arterial pressure
- □ Increased or decreased pulse rate
- □ Decreased respiratory rate
- □ Apnea
- □ Headache
- □ Hiccoughs
- □ Nausea
- □ Emesis/vomiting

#### Haloperidol

**Generic name:** Haloperidol injection, USP

**Brand name:** n/a, DIN 00808652 (5 mg/mL, 1-mL ampule)

Haloperidol injection (IM) is a butyrophenone derivative with antipsychotic properties that has been considered particularly effective in the management of hyperactivity, agitation, and mania. Haloperidol is an effective neuroleptic and also possesses antiemetic properties; it has a marked tendency to provoke extrapyramidal effects and has relatively weak alpha-adrenolytic properties. It may also exhibit hypothermic and anorexiant effects, and potentiate the action of barbiturates, general anesthetics, and other CNS depressant drugs.

### Dosing and administration

The control group will receive a single IM injection of the combination 5 mg midazolam and 5 mg haloperidol administered by a study nurse under the investigator’s or sub-investigator’s direction.

Potential side effects:

- □ Prolongation of the QT_c_ interval
- □ Orthostatic hypotension
- □ Extrapyramidal symptoms (e.g. dystonias, akathisia, parkinsonism, tardive dyskinesia)
- □ Agitation
- □ Drowsiness, insomnia, sedation
- □ Hyperprolactinemia
- □ Priapism
- □ Anticholinergic effects (e.g. dry mouth, constipation, urinary retention)
- □ Tachycardia, ECG abnormalities
- □ Neuroleptic malignant syndrome

#### Study products supply and accountability procedures

Study medication will be purchased by Providence Health Care Pharmacy and stored at the study site in a secure, limited access medication storage system (Omnicell).

Study medication will be organized and labelled in the hospital pharmacy and delivered to the Omnicell storage system per standard hospital procedures. An unblinded ED study nurse will prepare and administer study medication after opening the next sequential envelope in the randomization system stored with the study medication. A medication dispensing log will be used to track and maintain accountability for the study medication. Unused study products will be disposed of according to manufacturers’ instructions. Study medications will be handled and stored according to instructions provided in the respective Product Monographs.

The Canadian Controlled Drugs and Substances Act (CDSA) regulates controlled and targeted substances possession, transport, and administration. The study site maintains a system for controlling access through facility and station-based locked storage and an audited reconciliation process. The study medications used in this study will be managed using this existing system.

### Rescue medications

Participants will receive rescue sedation medications at the discretion of the treating ED study investigator. Rescue medications are defined as benzodiazepines, antipsychotics, or other sedative medications prescribed by the treating emergency physician for the purpose of behavioral control.

## Risks and precautions

### Risk management

Risk minimization, management, and assessment procedures have been implemented in the study to minimize and assess potential risks to those who participate in this clinical study with ketamine, midazolam, and haloperidol. Components include: (1) specific study entry and exclusion criteria to ensure that participants who have underlying characteristics that potentially increase their risk for an adverse outcome are excluded; and (2) protocol-specific procedures for minimizing and managing AEs, such as no patients aged > 60 years or women who are known or suspected to be pregnant or breast feeding will be enrolled. Additionally, all participants will receive routine ED care for this patient population including: (i) basic laboratory testing; (ii) electrocardiogram; (iii) complete physical exam by a study ED physician; and (iv) vital signs collection and monitoring every 5 min; and (3) overview surveillance will be done by an independent Medical Monitor.

This study does not involve a Data Monitoring Committee (DMC). The decision to not form a DMC for this study was arrived at following external review by experienced emergency physician peer review, external peer review by Health Canada, and two independent grant funding agencies.

## Clinical and laboratory evaluations

### Clinical evaluations

The blinded treating ED study investigator and study nurse(s) caring for the participant will be asked to assess the level of sedation of the participant and record the number of minutes required to reach a RASS < − 1 (Additional file 1), following study medication administration. Observation will start immediately after study medication administration and every 5 min until 30 min post study medication administration. The participant’s outcomes will be treated as censored after 30 min. In addition, if administration of rescue medication is required during the 5–30 min period following study medication(s) administration, this information will be collected. Rescue medications are defined as benzodiazepines, antipsychotics, or other sedative medications prescribed by the treating ED study investigator for the purpose of achieving behavioral control.

The time required from first dose of study medication(s) administration until assessment by a psychiatric nurse or psychiatrist in the ED will be collected.

For each participant sedation outcomes will be collected based on the Tracking and Reporting Outcomes Of Procedural Sedation (TROOPS) criteria form. The TROOPS criteria form is a multidisciplinary, consensus-based, standardized quality improvement tool developed by the International Committee for the Advancement of Procedural Sedation. Refer to Additional file 1 and https://www.sciencedirect.com/science/article/pii/S0007091217539471.

A 100 mm visual analog scale (VAS; Additional file 1) will be utilized to measure dissociative/emergence reaction symptoms. The Barnes Akathisia Scale [30] is a rating scale for the assessment of drug-related akathisia. (Additional file 1) Scores are in the range of 0 (no drug-related akathisia) to 14 (severe drug-related akathisia). It will be used to measure the occurrence and severity of abnormal movements. The Global Dystonia Severity Rating Scale (GDS) is a rating scale for the assessment of dystonia in ten body areas with scores in the range of 0 (no dystonia) to 100 (severe dystonia) (Additional file 1) [31].

Neuroleptic malignant syndrome is a potential risk factor with administration of the study medications. Clinical evaluation for neuroleptic malignant syndrome will be conducted within 24 h following study medication(s) administration as well as the follow-up visit on day 3.

Routine ED care will be conducted as described in Table 1: Schedule of Events.

**Table 1:** Schedule of events

	Visit type	
	Screening and treatment visit	Follow-up visit^a^
Procedures:	Day 1	Day 3 (± 1 day)
Assessment of eligibility	X	
Randomization	X	
Study drug administration	X	
Vital signs^b^	X	
Complete physical exam	X	
Routine laboratory testing^c^	X	
ECG	X	
RASS^b^	X	
Time to sedation	X	
Time to psychiatric assessment (in the ED)	X	
Rescue medication assessment^b^	X	
Incidence of sedation outcomes	X	
Use of physical restraints	X	
Effectiveness of Study Drug Blinding Survey	X	
Study Nurse Experience Survey	X	
Participant Experience Survey	X	
VAS	X	
Barnes Akathisia Scale	X	
GDS	X	
Pre-hospital use of force	X	
Adverse event collection	X	X
Neuroleptic malignant syndrome	X^d^	X
Review participant information sheet	X	

^a^Via telephone or in-person

^b^Conducted immediately after study medication administration and every 5 min until 30 min, after study medication administration

^c^CBC, rapid metabolic panel, toxicology screen

^d^Assessed within 24 h following study medication administration

### Surveys

For each participant, the blinded study investigator or other staff member will be asked to provide their personal impression of which medication the participant received at 30 min following medication administration, using the Effectiveness of Study Drug Blinding Survey (Additional file 1). Only one survey will be completed for each participant.

For each participant, the blinded ED study nurse administering the study medications will be asked to assess his/her perceptions of participant care using the Study Nurse Experience Survey (Additional file 1).

If feasible, study participants will be asked to assess their perception of their ED care experience using the Participant Experience Survey (Additional file 1) before being discharged from the ER.

## Study procedures

### Screening and treatment visit procedures

**Fig. 1:**
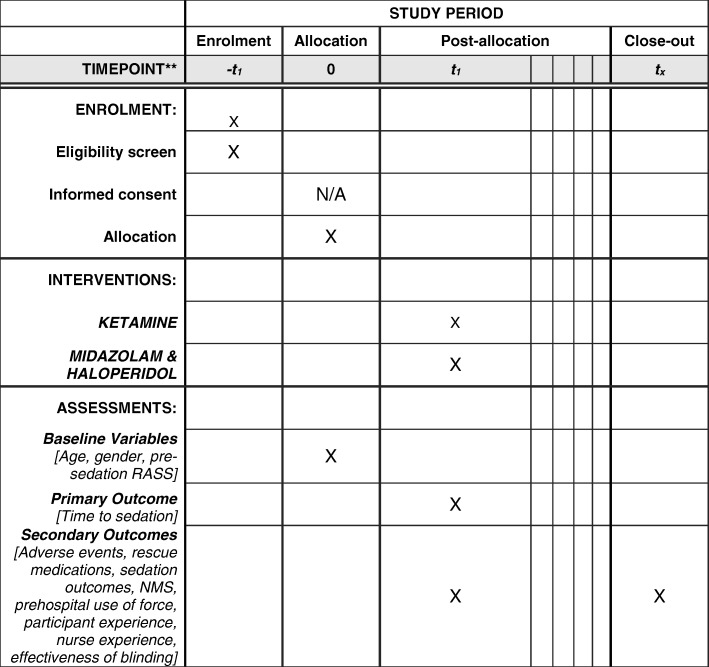
SPIRIT Flow Diagram of schedule of enrolment, interventions, and assessments

Designated study staff in the ED will identify potential participants, screen patients for eligibility, and conduct data collection. Please refer to (Fig. 1).

The treatment visit will occur immediately following pre-study screening procedures.

Following the patient’s assessment of eligibility, the following procedures will be conducted by the blinded study staff except where noted below:

- □ Enrollment and randomization;
- □ Study medication(s) administration (by the unblinded ED study nurse);
- □ Routine ED care for this patient population including:
  - ○ Basic laboratory testing (CBC, rapid metabolic panel, toxicology screen)
  - ○ Electrocardiograph
  - ○ Complete physical exam, including estimated weight
  - ○ Vital signs collection and monitoring immediately after study medication administration and every 5 min until 30 min after medication administration;
- □ Assessment of the level of sedation; time required to reach a RASS < − 1 (Additional file 1);
- □ Assessment of the time (in minutes) required from first dose of study medication(s) administration until assessment by psychiatric nurse or psychiatrist in the ED;
- □ Administration of rescue medication; as required;
  - ○ Assessment of rescue medications between 5 and 30 min (at 5-min intervals) of initial IM medication administration. Rescue medications are defined as benzodiazepines, antipsychotics, or other sedative medications prescribed by the treating emergency physician for the purpose of achieving behavioral control;
- □ Use of physical restraints (including duration of application);
- □ Incidence of sedation outcomes as defined by the TROOPS criteria (Additional file 1);
- □ Effectiveness of Study Drug Blinding Survey (Additional file 1);
- □ Study Nurse Experience Survey (Additional file 1);
- □ Participant Experience Survey (Additional file 1);
- □ VAS for dissociative/emergence reaction symptoms (Additional file 1);
- □ Barnes Akathisia Scale for occurrence and severity of abnormal movements (Additional file 1);
- □ GDS for occurrence and severity of dystonia (Additional file 1);
- □ Pre-hospital use of force (number and type(s) of restraint);
- □ Collection of AEs;
- □ Assessment for neuroleptic malignant syndrome within 24 h following study medication(s) administration;
- □ Review of Participant Information Sheet and discussion with participant.

All rescue medications and SAEs will be recorded as part of regular study data collection. All AEs will be followed up until their resolution or stabilization; refer to Section 10.4.

Standard ED tests and interventions for these patients will proceed without interruption from the study. Study participants will continue to receive the standard of care treatment if presenting with agitated and violent behavior.

### Follow-up visit procedures

The follow-up visit will occur on day 3 (± 1 day) via telephone or in-person. The following procedures will be conducted:

- □ Assessment for neuroleptic malignant syndrome;
- □ Collection of AEs.

## Evaluation, recording, and reporting of adverse events

### Definitions

#### Adverse events

An AE is any untoward medical occurrence in a patient or clinical investigation participant, administered a study medication/intervention, which does not necessarily have a causal relationship with this treatment. An AE can therefore be any unfavorable and unintended sign (including an abnormal laboratory finding), symptom, or disease temporally associated with the use of a medicinal (investigational) study medication/intervention, whether or not related to the medicinal (investigational) study medication/intervention.

During the treatment and safety follow-up visits, information on AEs will be gathered and documented accordingly. AEs will be graded as mild, moderate, severe, or life-threatening and assessed by causality as probably related, possibly related, unlikely to be related, or not related to the study medications.

Stable chronic conditions which are present before clinical trial entry and do not worsen are not considered AEs and will be accounted for in the participant’s medical history.

#### Serious adverse events

An SAE is defined as an AE meeting one of the following criteria at any dose:

- □ Results in death during the period of protocol-defined surveillance;
- □ Is a life-threatening event (defined as a participant at immediate risk of death at the time of the event);
- □ Results in inpatient hospitalization or prolongation of existing hospitalization during the period of protocol-defined surveillance;
- □ Results in persistent or significant disability or incapacity (disability is defined as a substantial disruption of a person’s ability to conduct normal life functions);
- □ Is a congenital anomaly or birth defect.

Any other important medical event that may not result in one of the above outcomes, may be considered a SAE when, based upon appropriate medical judgment, the event may jeopardize the participant and may require medical or surgical intervention to prevent one of the outcomes listed above. Examples of such medical events include allergic bronchospasm requiring intensive treatment in an emergency room or at home, blood dyscrasias or convulsions that do not result in inpatient hospitalization, or the development of drug dependency or drug abuse.

It is anticipated that patients included in this study will present to the ED with underlying medical issues that will require admission to hospital for stabilization and treatment (i.e. acute mental health crises, infections, drug overdoses, trauma). Inpatient hospitalization or admission to the Intensive Care Unit (ICU) will not be considered a SAE when the patient’s final discharge diagnosis from their hospital stay is attributed to these underlying medical conditions.

We will engage a Medical Monitor (independent ED physician not involved in the direct conduct of the study) to review SAEs as they occur.

### AE descriptions and recording

#### Intensity

The intensity (severity) for each AE (including SAE) will be graded according to the Common Terminology Criteria for Adverse Events (CTCAE) Version 4.0 (https://evs.nci.nih.gov/ftp1/CTCAE/About.html).

#### Relationship to study treatment

For all collected AEs (including SAEs), the clinician who examines and evaluates the participant will determine the AE’s causality based on temporal relationship and his/her clinical judgment. The degree of certainty about causality will be graded using the categories below:

- *Definitely related:* There is clear evidence to suggest a causal relationship, and other possible contributing factors can be ruled out. The clinical event, including an abnormal laboratory test result, occurs in a plausible time relationship to drug administration and cannot be explained by concurrent disease or other drugs or chemicals. The response to withdrawal of the drug (de-challenge) should be clinically plausible. The event must be pharmacologically or phenomenologically definitive, with use of a satisfactory re-challenge procedure if necessary;
- *Probably related:* There is evidence to suggest a causal relationship; the influence of other factors is unlikely. The clinical event, including an abnormal laboratory test result, occurs within a reasonable time sequence to administration of the drug, is unlikely to be attributed to concurrent disease or other drugs or chemicals, and follows a clinically reasonable response on withdrawal (de-challenge). Re-challenge information is not required to fulfill this definition;
- *Possibly related:* There is some evidence to suggest a causal relationship (e.g. the event occurred within a reasonable time after administration of the trial medication). However, the influence of other factors may have contributed to the event (e.g. the participant’s clinical condition, other concomitant events). Although an adverse drug event may rate only as “possibly related” soon after discovery, it can be flagged as requiring more information and later be upgraded to “probably related” or “definitely related,” as appropriate.
- *Unlikely:* A clinical event, including an abnormal laboratory test result, whose temporal relationship to drug administration makes a causal relationship improbable (e.g. the event did not occur within a reasonable time after administration of the trial medication) and in which other drugs or chemicals or underlying disease provides plausible explanations (e.g. the participant’s clinical condition, other concomitant treatments).
- *Not related:* The AE is completely independent of study drug administration and/or evidence exists that the event is definitely related to another etiology. There must be an alternative, definitive etiology documented by the clinician.

#### Reporting and evaluation of SAEs and other clinically significant AEs

##### SAEs

All SAEs that occur during the course of the study must be reported to the Sponsor-Investigator within 24 h of the site becoming aware of the event. The Centre for Health Evaluation and Outcome Sciences (CHÉOS) will be responsible for reporting expedited events to Health Canada on behalf of the Sponsor-Investigator within the required timeframe.

##### Follow-up for adverse events

All AEs (including SAEs) will be followed where possible until resolution or until the investigator has determined that the AE has resolved, stabilized, or become chronic and no further follow-up is required.

## Statistical considerations

### Sample size considerations/justification

This is the first randomized double-blind controlled trial on the use of ketamine for rapid, primary behavioral control in the ED. Previous work on this topic has demonstrated a mean time to sedation of 7 min for patients receiving the benzodiazepines and antipsychotics. The projected mean time of sedation for patients receiving ketamine in this study is anticipated to be 4 min (unpublished data), resulting in a mean reduction of 3 min and a hazard ratio (HR) of 7/4 = 1.75. However, to ensure adequate power, the sample size calculation will assume a lower mean reduction of 2.5 min (HR = 7/4.5 = 1.56).

To achieve 80% power, assuming a HR =1.56, an alpha = 0.05, and patients are followed up to 30 min, the required sample size is 166 (83 per group). Assuming a 10% loss to follow up [attrition], a final sample size of 184 (92 patients per group) will be required. If indeed the true mean time to sedation for patients receiving ketamine is 4 min (3 min reduction), the power of this trial will be 94%.

### Datasets to be analyzed

Data collected during the study will be entered into a designed database. Data will be checked by a data manager and any queries will be resolved with the assistance of study staff.

The finalized datasets to be analyzed will be imported into statistical software, e.g. SAS system. All the datasets will include a unique identifier for each patient. The structure of each dataset is to have one patient per row and one variable per column.

### Outcome measures

Primary endpoint: Time from first IM medication administration to adequate sedation which is defined as RASS ≤ − 1.

Secondary endpoints:

1. Percentage participants with adverse events in each arm;
2. Percentage of participants in each arm requiring rescue medications between 5 and 30 min (at 5-min intervals) after study medication(s) administration by count;
3. Percentage of participants in each arm experiencing sedation outcomes as defined by the TROOPS criteria;
4. Percentage of participants with neuroleptic malignant syndrome events within 24 h of enrollment of each arm;
5. Percentage of participants experiencing pre-hospital use of force by police in this participant population, by number and type of restraint;
6. Participant experience survey outcomes;
7. Study Nurse Experience Survey outcomes;
8. Effectiveness of Blinding Survey outcomes.

### Analysis of primary outcome measures

A Cox proportional hazards model will be used to assess the difference in time to adequate sedation between the two arms. In the analysis, patients who do not achieve adequate sedation criteria will be censored at the end of follow-up period of 30 min. The model will adjust for the following covariates: age; gender; and RASS before administration of study medication. The hazard ratio between the two arms and associated 95% confidence interval (CI) will be reported.

Analysis will be based on an intent-to-treat basis, i.e. the patient will be included in the analysis as being randomized. An as-treated analysis will be also conducted as a sensitivity analysis.

### Analysis of secondary outcome measures

AEs will be summarized for each arm by presenting the number and percentage of participants having any AEs, having AEs in severity levels (graded as mild, moderate, severe, or life-threatening). The associated 95% CI of the percentage will also be reported.

Participants requiring rescue medications between 5 and 30 min of initial IM medication administration in each arm will be summarized as percentage, along with its 95% CI.

The number and percentage of participants with any sedation outcomes will be summarized for each arm, along with the 95% CI of the percentage. Furthermore, the number and percentage in each type of sedation outcomes will be presented.

Incidence of neuroleptic malignant syndrome within 24 h of enrollment of each arm will be summarized as count and percentage and associated 95% CI.

Percentage of participants experiencing pre-hospital use of force by police in the cohort will be reported as well as its 95% CI. Furthermore, the number and percentage in each type of restraints will be presented.

Participant’s clinical experience survey is a questionnaire with four questions (regarding feeling on medication and sedation medications) and answered as YES/NO. The number and percentage (and its 95% CI) of patients chosen YES (or NO) for each question will be reported.

Nurse’s clinical experience survey is a questionnaire with four questions (regarding feeling on medication and sedation medications) and answered as YES/NO. The number and percentage (and its 95% CI) of study nurse chosen YES (or NO) for each question will be reported.

To evaluate the effectiveness of blindness of the study, study nurses and physicians will be asked to guess the medication (Midazolam and haloperidol or Ketamine or “don’t know”) that the participant received at the end of the study. A table of guess versus treatment received will be generated for the answers from study nurses and as well physicians. Blinding indices and associated 95% CI will be reported.

### Other variables of interest

Participant demographic and clinical characteristics will be collected and summarized as mean and standard deviation (SD) or median and interquartile range for continuous variables and count and percentage for categorical variables as well as its 95% CI. Demographic variables include: age and gender. Clinical variables of interest include: (1) RASS before IM administration; (2) a VAS to measure dissociative/emergence reaction symptoms; 3) Barnes Akathisia Scale to measure occurrence and severity of abnormal movements; 4) Global Dystonia Scale; 5) and percentage of participants with the use of physical restraints and duration of application.

## Ethical considerations

This study will be conducted in accordance with the ICH GCP Guidelines, Tri-Council Policy statement, and the principles in the Declaration of Helsinki. The Investigator will be thoroughly familiar with the appropriate use of the study treatment as described in the protocol and study medication Product Monographs.

### Informed consent

A waiver for informed consent has been granted by the UBC Providence Health Care Research Ethics Board (PHC REB) for this study.

### Confidentiality

All participant-related information including CRFs, evaluation forms, reports, etc. will be kept strictly confidential. All records will be kept in a secure, locked location and only accessible to research staff. Participants will be identified only by means of a coded number specific to each participant. All computerized databases will identify participants by numeric codes only and will be password-protected.

Upon request, and in the presence of the investigator or his/her representative, participant records will be made available to the study sponsor, monitoring groups representative of the study sponsor, representatives of funding groups, and applicable regulatory agencies for the purpose of verification of clinical trial procedures and/or data, as is permissible by local regulations.

Participants will be assigned a unique study identifier code to maintain confidentiality during and after the research study. Participants will be identified on data collection forms by this unique identifier.

## General trial conduct considerations

### Adherence to protocol

#### Protocol amendments

All protocol amendments will be reviewed and approved and if applicable submitted to the applicable regulatory agencies for prior approval or notification. The Investigator must sign and date the amendment before implementation. All protocol amendments must also be submitted to the ethics committee.

#### Protocol deviations

No deviations from this protocol will be permitted without the prior written approval of the Sponsor-Investigator, except when the modification is needed to eliminate an immediate hazard or hazards to participants. Any deviations that may affect a participant’s treatment or informed consent, especially those increasing potential risks, must receive prior approval from the REB unless performed to remove an immediate safety risk to the participants. In this case it will be reported to the REB and the Sponsor-Investigator immediately thereafter. Any departures from the protocol must be documented.

#### Study monitoring

The study site agrees to allow study monitors from the Sponsor-Investigator and/or his representatives direct access to the study records and medical records from those patients enrolled in the clinical study as well as drug accountability records. Adequate monitoring space and time must be provided. The Sponsor-Investigator representative will perform ongoing study site monitoring after the first 10–15 participants have been enrolled and then at random intervals during enrolment to ensure compliance.

Protocol deviations will be monitored and recorded by the study monitor.

### Record keeping

#### Source documents

The Investigator must maintain adequate and accurate source documents upon which CRFs for each participant are based. They are to be separate and distinct from CRFs except for cases in which the Sponsor-Investigator has pre-determined that direct data entry into specified pages of the participant’s CRF is appropriate. These records should include detailed notes on:

- □ Inclusion and exclusion criteria details;
- □ RASS;
- □ AEs and concomitant medication;
- □ Results of relevant examinations;
- □ Laboratory printouts;
- □ Enrolment number;
- □ Compliance/non-compliance protocol deviation information.

### Data management

Instructions concerning the recording of study data on electronic CRFs will be provided by the CHÉOS Data Management Centre to assure the quality of computerized data for this study. The study site is responsible for submitting the data in a timely fashion. All data will be maintained on secure computer servers at CHÉOS.

### Record retention

The Investigator will maintain all study records according to the ICH-GCP and applicable regulatory requirement(s). Records will be retained for 25 years, in accordance with applicable regulatory requirement(s). If the Sponsor-Investigator withdraws from the responsibility of keeping the study records, custody must be transferred to a person willing to accept the responsibility. No destruction of medical records should occur without the written approval of the Sponsor-Investigator.

All data will be destroyed by trained IT professionals after 25 years.

## Discussion section

We present the protocol for a novel study to determine whether ketamine is a rapid and safe option, compared to a combination of midazolam and haloperidol, for the sedation of patients presenting to the ED with agitation and violent behavior. To our knowledge, this study is the first randomized controlled trial to compare ketamine to current standard care for this indication.

We have attempted to address numerous logistical issues with the design of this study. Patients presenting to the ED with psychomotor agitation and violent behavior lack the capacity to provide informed consent for research purposes and the lack the ability for the informed refusal of medical care. As a result, we have obtained a waiver of informed consent from our institutional Research Ethics board. In cases where a potential participant presents to the ED accompanied by an appropriate substitute decision-maker, informed consent will be obtained from the appropriate substitute decision-maker. We feel that this strikes a fine balance between the ethical principles of respect for patient autonomy and non-maleficence, and safely conducting research on a pressing issue of patient safety in a marginalized group of patients lacking the capacity to provide informed consent or informed refusal of care.

The pharmacodynamic effects of ketamine for procedural sedation provide a unique challenge for the conduct of this study. Prior work has demonstrated that ocular nystagmus is a key physical exam finding that can distinguish between patients receiving ketamine compared to midazolam [32]. To maintain adequate blinding of the data collectors and outcome assessors for this study, they will remain behind opaque dividers in the clinical care space in the ED to prevent them from observing potential ocular nystagmus in the intervention group, or the lack of nystagmus in the control group.

Timely participant enrolment of eligible patients can be a challenge with any randomized controlled trial. This is a single-center study and our required sample size is 184 patients. To ensure adequate and timely patient enrolment, we will endeavor to have study staff members present in the ED approximately 16 h per day, each day of the week. Our site sees approximately three potentially eligible patients each day and we anticipate being able to reliably enroll approximately one patient per day, allowing for complete enrolment in approximately 6–8 months. However, it is possible that patient enrolment may not occur as efficiently as anticipated. We will consider expanding this from a single-center to multi-center study if the enrolment of patients is suboptimal at approximately eight months into the trial.

Another anticipated challenge of this trial is that this a marginalized and high-risk population with high rates of substance use, mental health challenges, and social marginalization, including homelessness. This patient population may not be compliant with all study requirements. We hypothesize that the rate of completion of the Patient Experience Survey will be low. Further, we anticipate that compliance rates will be very low for the follow-up visit at approximately three days after the index visit.

This study is being supported and monitored by the Centre for Health Evaluation and Outcome Science (CHEOS) at St Paul’s Hospital, Vancouver, Canada. CHEOS has extensive expertise and experience in the conduct of clinical trials, data monitoring and management, data analysis, and regulatory compliance in the areas of HIV, substance use, and marginalized populations. We are confident that this support capacity will allow for active monitoring of data quality and completeness, regulatory compliance, and the rapid assessment of AEs and SAEs (Additional file 2).

## Trial status

The protocol reported here is version 2.2, dated 18 December 2017. Trial enrolment began on 11 June 2018 and is anticipated to finish on 30 June 2019.

## Additional file

Additional file 1: Richmond Agitation Sedation Scale (RASS). TROOPS Criteria. Effectiveness of Study Blinding Survey. Study Nurse Experience Survey. Participant Experience Survey. Visual Analog Scale for Emergence Reactions. Barnes Akathisia Scale. Global Dystonia Severity Rating Scale. (DOCX 400 kb)

Additional file 2: SPIRIT 2013 Checklist: Recommended items to address in a clinical trial protocol and related documents*. (DOC 121 kb)

## Abbreviations

AE: Adverse event
CDSA: Controlled Drug and Substance Act
CRF: Case report form
ED: Emergency department
GDS: Global Dystonia Severity Rating Scale
ICH GCP: International Conference on Harmonisation Good Clinical Practice
IM: Intramuscular
NMDA: N-methyl-D-aspartate
RASS: Richmond Agitation-Sedation Scale
REB: Research Ethics Board
SAE: Serious adverse event
TROOPS: Tracking and Reporting Outcomes of Procedural Sedation
VAS: Visual analog scale
