# Supplementary material for: Rapid agitation control with ketamine in the emergency department (RACKED): a randomized controlled trial protocol
Source: Trials. 2018 Nov 26;19:651. doi: 10.1186/s13063-018-2992-x (PMC6258312; doi:10.1186/s13063-018-2992-x)
Supplement: Supplementary file 1 — Richmond Agitation Sedation Scale (RASS). TROOPS Criteria. Effectiveness of Study Blinding Survey. Study Nurse Experience Survey. Participant Experience Survey. Visual Analog Scale for Emergence Reactions. Barnes Akathisia Scale. Global Dystonia Severity Rating Scale. (DOCX 400 kb) [file 13063_2018_2992_MOESM1_ESM.docx]

## **Richmond Agitation Sedation Scale (RASS)**

**
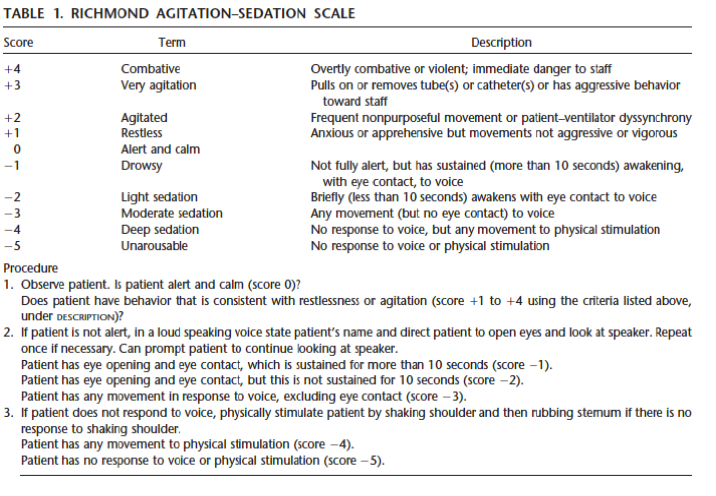
**

## **TROOPS Criteria**

Refer to <http://proceduralsedation.org/TROOPS.html>

**
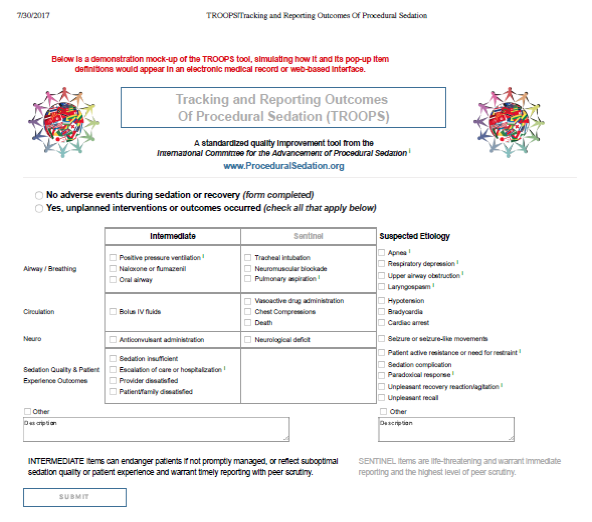
**

## **Effectiveness of Study Drug Blinding Survey**

Please choose the study medicine(s) that you think the participant received:

- 1. Midazolam and haloperidol
  2. Ketamine
  3. Don’t Know

## **Study Nurse Experience Survey**

*(all YES/NO answers)*

1. Do you feel this participant received adequate sedation?
2. Did the sedation occur fast enough?
3. Was the study medication too difficult to administer?
4. Were you concerned about participant’s safety giving this medication?

## **Participant Experience Survey**

*(all YES/NO answers)*

1. Do you feel the medicine(s) you received in the emergency department were safe?
2. Do you have any bad memories or bad dreams from receiving the medicines that you did?
3. If you have received sedation medicine(s) in the emergency department before, did this time feel better?
4. If you have received sedation medicine(s) in the emergency department before, did this time feel worse?

## **Visual Analog Scale for Emergence Reactions**

No Emergence Reaction Severe Emergence Reaction

**0 100**

**________________________________________________________________**

## **Barnes Akathisia Scale**

**1. Objective SCORE: _____**

1. Normal, occasional fidgety movements of limbs.
2. Presence of characteristic restless movements: shuffling or tramping movements of the legs/feet or swinging of one leg while sitting, *and/or* rocking from foot to foot or ‘walking on the spot’ when standing, *but* movements present for less than half the time observed.
3. Observed phenomena, as described in (1) above, which are present for at least half the observation period.
4. Patient is constantly engaged in characteristic restless movements, *and/or* has the inability to remain seated or standing without walking or pacing, during the time observed.

**2. Subjective: Awareness of restlessness SCORE: _____**

1. Absence of inner restlessness.
2. Non-specific sense of inner restlessness.
3. Patient is aware of an inability to keep the legs still, or a desire to move the legs, and/or complains of inner restlessness aggravated specifically by being required to stand still.
4. Awareness of an intense compulsion to move most of the time and/or reports a strong desire to walk or pace most of the time.

**3. Subjective: Distress related to restlessness SCORE: _____**

1. No distress.
2. Mild.
3. Moderate.
4. Severe.

**4. Global clinical assessment of akathisia SCORE: _____**

1. Absent.

No evidence of awareness of restlessness. Observation of characteristic movements of akathisia in the absence of a subjective report of inner restlessness or compulsive desire to move the legs should be classified as pseudoakathisia.

1. Questionable.

Non-specific inner tension and fidgety movements.

1. Mild akathisia.

Awareness of restlessness in the legs and/or inner restlessness worse when required to stand still. Fidgety movements present, but characteristic restless movements of akathisia not necessarily observed. Condition causes little or no distress.

1. Moderate akathisia.

Awareness of restlessness as described for mild akathisia above, combined with characteristic restless movements such as rocking from foot to foot when standing. Patient finds the condition distressing.

1. Marked akathisia.

Subjective experience of restlessness includes a compulsive desire to walk or pace. However, the patient is able to remain seated for at least five minutes. The condition is obviously distressing.

1. Severe akathisia.

The patient reports a strong compulsion to pace up and down most of the time. Unable to sit or lie down for more than a few minutes. Constant restlessness which is associated with intense distress and insomnia.

## **Global Dystonia Severity Rating Scale**

The global score is an overall score for the body area. The investigator rates the participant in relationship to all participants. If the dystonia changes during the examination, the rating for the maximal dystonia is recorded.

| 1. **Body Area** | **0**  **(No dystonia present)** | **1**  **(Minimal dystonia)** | **5**  **(Moderate dystonia)** | **10**  **(Most severe dystonia)** |
| --- | --- | --- | --- | --- |
|  | *Check only one* | | | |
| - - - 1. Eyes and upper face | ⬜ | ⬜ | ⬜ | ⬜ |
| - - - 1. Lower face | ⬜ | ⬜ | ⬜ | ⬜ |
| - - - 1. Jaw and tongue | ⬜ | ⬜ | ⬜ | ⬜ |
| - - - 1. Larynx | ⬜ | ⬜ | ⬜ | ⬜ |
| - - - 1. Neck | ⬜ | ⬜ | ⬜ | ⬜ |
| - - - 1. Shoulder and proximal arm | ⬜ | ⬜ | ⬜ | ⬜ |
| - - - 1. Distal arm and hand including elbow | ⬜ | ⬜ | ⬜ | ⬜ |
| - - - 1. Pelvis and upper leg | ⬜ | ⬜ | ⬜ | ⬜ |
| - - - 1. Distal leg and foot | ⬜ | ⬜ | ⬜ | ⬜ |
| - - - 1. Trunk | ⬜ | ⬜ | ⬜ | ⬜ |
